# Prevalence of extensively drug-resistant gram negative bacilli in surgical intensive care in Egypt

**DOI:** 10.11604/pamj.2014.19.177.4307

**Published:** 2014-10-21

**Authors:** Ahmed Hasanin, Akram Eladawy, Hossam Mohamed, Yasmin Salah, Ahmed Lotfy, Hanan Mostafa, Doaa Ghaith, Ahmed Mukhtar

**Affiliations:** 1 Department of anesthesia, Faculty of Medicine, Cairo University, Cairo, Egypt; 2 Department of clinical pathology, Faculty of Medicine, Cairo University, Cairo, Egypt

**Keywords:** Extensive drug resistance, gram negative bacilli, critically ill patients

## Abstract

**Introduction:**

The prevalence of extensively drug resistant gram negative bacilli (XDR-GNB) is rapidly progressing; however in Egypt data are sparse. We conducted the present study to quantify the incidence, risk factors and outcome of patients harboring XDR-GNB.

**Methods:**

A one year prospective study was done by collecting all the bacteriological reports for cultures sent from the surgical intensive care unit, Cairo university teaching hospital. XDR-GNB were defined as any gram negative bacilli resistant to three or more classes of antimicrobial agents. Patients with XDR-GNB compared with those sustaining non extensively drug-resistant infection. A multivariate logistic regression model was created to identify independent predictors of multi-resistance.

**Results:**

During one-year study period, a total of 152 samples (65%) out of 234 gram negative bacilli samples developed extensively drug resistant infection. XDR strains were significantly higher in Acinetobacterspp (86%), followed by Pseudomonas (63%), then Proteus (61%), Klebsiella (52%), and E coli (47%). Fourth generation cephalosporine (Cefipime) had the lowest susceptibility (10%) followed by third generation cephalosporines (11%), Quinolones (31%), Amikacin (42%), Tazobactam (52%), Carbapinems (52%), and colistin (90%). Relaparotomy was the only significant risk factor for acquisition of XDR infection.

**Conclusion:**

Extensively drug-resistant gram negative infections are frequent in our ICU. This is an alarming health care issue in Egypt which emphasizes the need to rigorously implement infection control practices.

## Introduction

Infection due to gram negative bacilli is a growing problem in clinical practice especially in tertiary medical centers. Patients in the intensive care unit (ICU) are highly vulnerable to infections because of impaired host defenses, administration of some drugs e.g. muscle relaxant, and the use of invasive devices that bypass the normal anatomical barriers. The growing emergence of resistant pathogens is considered an additional burden and a major threat to public health [1]. ICU is claimed to be the epicenter and the factory for rising, development and amplification of antimicrobial resistance. Although, there are currently no internationally accepted definition for the resistant microorganism, the term extensively-drug resistance (XDR) is used to denote isolates resistant to all but one or two classes of antimicrobial agents.

Surveillance studies are not only useful in detection of the prevalence of XDR organisms and taking infection control alarms, but also help determine the special pattern of antibiotic resistance to reach higher rates of initial appropriate therapy as well as saving last-line antibiotic agents [2]. Although some authors reported the prevalence and the burden of nosocomial infections in developing countries in terms of mortality, cost, and length of stay [3]; the data addressing prevalence of XDR is scarce in Egypt, we conducted a prospective cohort study to quantify the incidence, risk factors, and outcome of patients harboring XDR gram negative bacilli in our surgical intensive care unit.

## Methods

A one year prospective study was conducted in the Surgical Intensive Care Unit (SICU), emergency department of general Surgery, Cairo University hospitals Cairo university teaching hospital is the largest tertiary hospital in Egypt with 5500 bed capacity. All Patients who had gram negative bacilli isolates recovered from clinical cultures within the first 48 h after ICU admission with clinical signs of sepsis which led to the use of antibiotics directed against the organism(s) isolated; otherwise the patient was considered to be colonized with the organism. Colonization cases, defined as any positive culture without clinical signs of infection, were excluded. Patients who did not sustain an infection also were excluded. Cultures showing more than 3 isolates were excluded. In addition, all infections that occurred before patients’ admission to the SICU, within the first 48 hr of SICU hospitalization, or after 48 h after their discharge were also excluded. 281 consecutive isolates recovered from clinical specimens, including blood, urine, wound/tissue, and respiratory specimens (one pathogen per cultured site per patient) of ICU patients. The 281 isolates obtained represented 230 patients, anaerobic and fungal organisms were excluded. Isolates were shipped to the reference laboratory (Health Sciences Centre, Winnipeg, Canada) on Amiescharcoal swabs, subcultured onto appropriate media.

### Data collection

The following data were recorded from all patients with positive results; age, sex, primary diagnosis, APACHE II score, prior antimicrobial therapy within last 3 months, septic shock, repeated surgical intervention, mechanical ventilation, and ICU length of the stay, presence of central venous lines, parenteral nutrition, renal replacement therapy, blood transfusion, reoperation and hospital mortality. Antibiotic susceptibilities were determined by the disc diffusion method (Bio-Rad, Marnes la Coquettes, France) according to the recommendations of the Antibiogram Committee of the French Microbiology Society (CA-SFM). The bacteria were then classified as susceptible or resistant (resistant and intermediate). The susceptibility of aerobic Gram negative bacteria to 10 antibiotics (cefoperazone, cefotaxime, ceftazidime, cefepime, cefoperazone-sulbactam, imipenem, gentamicin, amikacin, colistin and ciprofloxacin) was reported. Extensive drug resistant (XDR) gram negative bacilli were defined as isolates resistant to three or more groups of antimicrobials. Prevalence of XDR isolates was reported among different isolate sites as well as different type of organism. Patients were further divided into XDR and non XDR groups, both groups were compared as regards possible risk factors as well as outcome.

### Statistical analysis

Categorical variables were analyzed using the X2 or Fischer Exact test as appropriate. For continuous variables, the data are presented as median (range) and were analyzed with Mann-Whitney test. Parameters selected by univariate analysis to have P values < 0.10 were evaluated in the multivariate analysis. Risk factors independently associated with acquisition of XDR gram negative bacilli were identified by stepwise logistic regression analysis of variables selected by univariate analysis. The software SPSS v15.0 for Windows (SPSS, Inc, Chicago, Il, United States) was used for statistical analysis.

## Results

### Prevalence of XDR isolates

**Figure 1:**
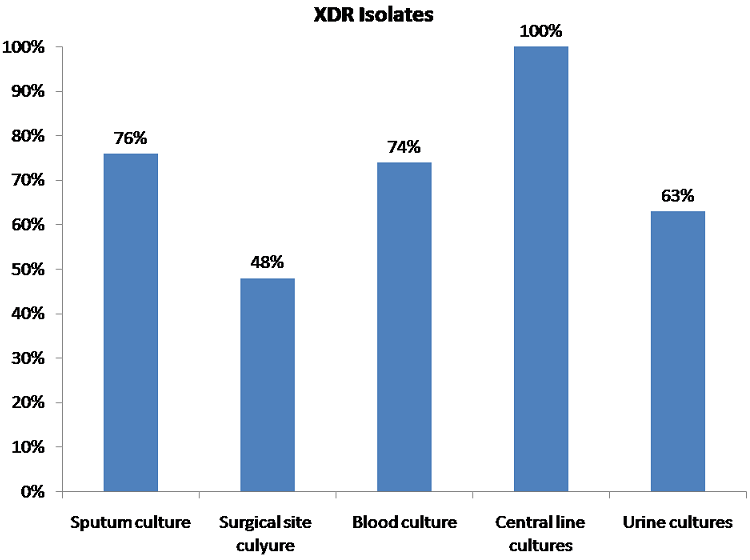
Prevalence of XDR strains among different sites of isolates. P value = 0.003

During one-year study period, a total of 152 samples (65%) out of 234 gram negative bacilli samples developed extensively drug resistant infection. XDR strains were significantly higher in Acinetobacterspp (86%), followed by Pseudomonas (63%), then Proteus (61%), Klebsiella (52%), and E coli (47%) (Table 1). As regards different sites of isolates, XDR strains were 75% of sputum cultures, 74% of blood cultures, 63% of urine cultures, 48% of surgical site cultures, and 100% of central line cultures (Figure 1).

**Table 1:** Prevalence of XDR isolates among different organisms

Isolate	Number of cultures (%)	XDR strains (%)
P. aeruginosa	60	63%
K. pneumonia	62	52%
A. baumannii	54	86%
E. coli	34	47%
P. mirabilis	13	61%

### Antimicrobial susceptibility

The susceptibility rates for different antimicrobial agents in our critically ill patients is shown in Table 2 where the fourth generation cephalosporine (Cefipime) had the lowest susceptibility (10%) followed by third generation cephalosporines (11%), Quinolones (31%), Amikacin (42%), Tazobactam (52%), Carbapinems (52%), and colistin (90%).

**Table 2:** Antibiotic susceptibility pattern of the most five abundant gram negative isolates

	C3	C4	Quinolones	Amikacin	Tazocin	Carbapinems	Colistin
P. aeruginosa	13%	16%	33.9%	38.3%	68.8%	39%	87.5%
A. baumannii	3.7%	2%	18.5%	17%	20%	24.5%	100%
K. pneumonia	11.3%	10.5%	45.9%	45.8%	35.7%	88.7%	100%
E. coli	11.8%	9.7%	39.4%	69.7%	66.7%	97.1%	100%
P. mirabilis	27.3%	30%	41.7%	36.4%	0%	83.3%	100%

C3: 3^rd^ generation cephalosporines, C4: 4^th^ generation caphalosporines

### Risk factors and outcome

Relaparotomy was the only risk factor reported to be associated with increased XDR infections by both univariate and multivariate analysis, all other risk factors included in the study were not significantly associated with acquisition of XDR (Table 3).

**Table 3:** Demographic data, risk factors, and outcome of MDR and Non MDR groups of patients (Univariate analysis). Data are represented as mean (SD) and frequency

	XDR group (n = 152)	Non XDR group (n = 82)	P value
Prevalence	65%	35%	
Age	36(±31)	30(±19)	
APACHE II	19(±9)	23(±10)	
Gender			0.4
Male	62.9%	67.9%	
Female	37.1%	32.1%	
Ventilation	84.7%	79%	0.3
Relaparotomy	55%	25%	0.04*
Previous sepsis	58.1%	55.9%	0.12
Mortality	57%	42.9%	0.07

* Denotes statistical significance

## Discussion

The main finding in our study was the high prevalence of XDR gram negative bacilli among all our isolates in all sites; this is similar to the findings of many studies all over the world [4–12]. Nosocomial infections carry a high burden in both developed and developing countries, this burden was reported in the terms of mortality, financial losses, and length of stay [1, 3]. although some studies reported the prevalence of XDR in Egypt [9–11], all of them were usually concerned with only one site of infection such as blood stream infections [9, 10] and lower respiratory tract infections [11], and only one study was concerned with critically ill patients in Egypt [9]. Our study described the prevalence of XDR gram negative bacilli in all sites of cultures among critically ill patients in a large teaching hospital.

Some studies reported lower prevalence of XDR than what we found [13, 14], this might be because they were conducted in the early 2000s [14] this is supported by the obvious increase of drug resistance year after year all over the world which was reported in few studies [15–18]. There was a unique exception reported in China by Qin Y et al [19] who described a decrease in XDR isolates, this decrease was explained by the adherence to the principles of antibiotic use and effective monitoring and preventive measures.

In our study the only significant risk factor for acquisition of XDR infection was relaparotomy while all other classic risk factors were not significantly associated with XDR infections. A similar finding was reported in a recent Belgian multicenter study which showed that some classic risk factors lost their predictive value as in 40% of infected patients with XDR microorganisms [20]. Seguin et al [21] reported a different finding where antimicrobial treatment in the 3 months preceding hospitalization, duration between first operation, and relaparotomy were shown to be independent risk factors for XDR in postoperative peritonitis, also Bayani el al [22] reported a correlation between the cause of hospitalization XDR Pseudomonas infection.

The main forces considered responsible for emergence and spread of XDR organisms are: 1) induction of resistant strains; 2) selection of resistant strains; 3) introduction of resistant strains; and 4) dissemination of resistant strains. These factors should be considered in the battle against the spread of antimicrobial resistance [23].

Prevalence of XDR isolates in our patients was higher in Pseudomonas and Acinetobacter species than other gram negative bacilli, this is similar to many studies that reported high antimicrobial resistance in these two organisms. Pseudomonas is intrinsically resistant to most antibiotics with multiple mechanisms that are responsible for Antimicrobial resistance such as hyperproduction of enzymes, such as beta-lactamases and DNA-gyrases, active efflux pumps, and permeability changes. Acinetobacter spp. are inheritably resistant to cephalosporins, penicillin's, and aminoglycosides, and especially cause opportunistic infections in critically ill patients. Some strains of A. baumannii have been detected that are resistant to all antibiotics [24, 25].

Having this high alarming prevalence of XDR infections needs aggressive strategies for infection control. The Center for Disease Control recommends four strategies for health care settings: 1) prevent infections; 2) diagnose and treat infections; 3) prudent and rational use of antimicrobials; and 4) prevent transmission [2, 26]. Another keystone in managing this problem is antibiotic stewardship to optimize the use of antimicrobials in ICU [27].

## Conclusion

In conclusion, extensively drug-resistant gram negative infections are endemic in our ICU. This is an alarming health care issue in Egypt which emphasizes the need to rigorously implement infection control practices.
